# Tumor-Infiltrating CD8 T Cells Predict Clinical Breast Cancer Outcomes in Young Women

**DOI:** 10.3390/cancers12051076

**Published:** 2020-04-26

**Authors:** Yong Won Jin, Pingzhao Hu

**Affiliations:** 1 Department of Biochemistry & Medical Genetics, Rady Faculty of Health Sciences, University of Manitoba, Winnipeg, MB R3E0J9, Canada; 2 Research Institute in Oncology and Hematology, Cancer Care Manitoba, Winnipeg, MB R3E 0V9, Canada; 3 Department of Electrical and Computer Engineering, Faculty of Engineering, University of Manitoba, Winnipeg, MB R3T 5V6, Canada; 4 Department of Computer Science, Faculty of Science, University of Manitoba, Winnipeg, MB R3T 2N2, Canada

**Keywords:** early-onset breast cancer, deconvolution, tumor infiltration lymphocytes, mutational signatures, pathway analysis

## Abstract

Young women with breast cancer have disproportionately poor clinical outcomes compared to their older counterparts. The underlying biological differences behind this age-dependent disparity are still unknown and warrant investigation. Recently, the tumor immune landscape has received much attention for its prognostic value and therapeutic targets. The differential tumor immune landscape between age groups in breast cancer has not yet been characterized, and may contribute to the age-related differences in clinical outcomes. Computational deconvolution was used to quantify abundance of immune cell types from bulk transcriptome profiles of breast cancer patients from two independent datasets. No significant differences in immune cell composition that were consistent in the two cohorts were found between the young and old age groups. Regardless of absence of significant differences, the higher tumor infiltration of several immune cell types, such as CD8+ T and CD4+ T cells, was associated with better clinical outcomes in the young but not in the old age group. Mutational signatures analysis showed signatures previously not found in breast cancer to be associated with tumor-infiltrating lymphocyte (TIL) levels in the young age group, whereas in the old group, all significant signatures were those previously found in breast cancer. Pathway analysis revealed different gene sets associated with TIL levels for each age group from the two cohorts. Overall, our results show trends towards better clinical outcomes for high TIL levels, especially CD8+ T cells, but only in the young age group. Furthermore, our work suggests that the underlying biological differences may involve multiple levels of tumor physiology.

## 1. Introduction

Breast cancer is the most prevalent type of cancer in women worldwide [1]. It is a highly heterogeneous disease with multiple subtypes and classifications [2]. There are disparities in pathological features and disease outcomes between younger (age at diagnosis <40) versus older (age at diagnosis ≥40) breast cancer patients [1,3,4]. Young women with breast cancer are more commonly diagnosed with aggressive, invasive types of breast cancer that are difficult to treat. Studies found survival to be inversely associated with age at diagnosis [4,5,6]. The underlying biological cause for this age-dependent disparity in survival outcome is still unknown [1].

Recent studies have provided accumulating evidence that the presence of tumor-infiltrating lymphocytes (TILs) and their composition show significant associations with prognosis and response to cancer treatments [7,8]. Specific TILs such as CD8+ cytotoxic T lymphocytes, T helper 1 cells, M1 macrophages, natural killer (NK) cells, and T-follicular helper (Tfh) cells have been reported as exhibiting anti-tumor activities, whereas T-regulatory (Treg) cells and M2 macrophages are known for their immune-inhibitory and thus pro-tumor activities [7]. Furthermore, measures of TILs have been shown to be markers for pathological complete remission, chemosensitivity, and improved recurrence-free survival, especially in non-luminal, receptor-negative breast cancers [9,10,11].

Today, there is an abundance of bulk transcriptomic data available publicly online [12,13,14]. These bulk transcriptomes often represent the average gene expression across a heterogeneous mixture of cells. If cellular components and their proportions can be identified from bulk transcriptomic data by computational methods, such in-silico methods can be used to characterize and quantify immune infiltrates in a cost-, time-, and labor-effective manner.

Deconvolution is a computational problem of simplifying a complex mixture into its individual constituents. In brief, most deconvolution algorithms see bulk transcriptome as a mixture where one gene of the mixture is a linear combination of that gene expressed across different cell types, weighted by the proportions of those cell types [15]. Deconvolution algorithms were previously applied to a large breast cancer transcriptomic dataset by others to characterize immune infiltrates across multiple samples [16,17]. After stratification by estrogen receptor (ER) status, Ali et al. found CD8+ T cells and activated memory T cells to be associated with favorable clinical outcomes in ER-negative tumors, which supported similar findings in the literature [7,16,18].

The tumor immune landscape, as predicted by computational deconvolution, has the potential to provide prognostic information as well as providing insight into immune functions within solid tumors. If age group differences in tumor immune landscape exist, these differences may be able to explain the age-dependent disparities in clinical outcomes in breast cancer.

## 2. Results

### 2.1. Estimates of Immune Cell Abundance by TIMER

**Figure 1:**
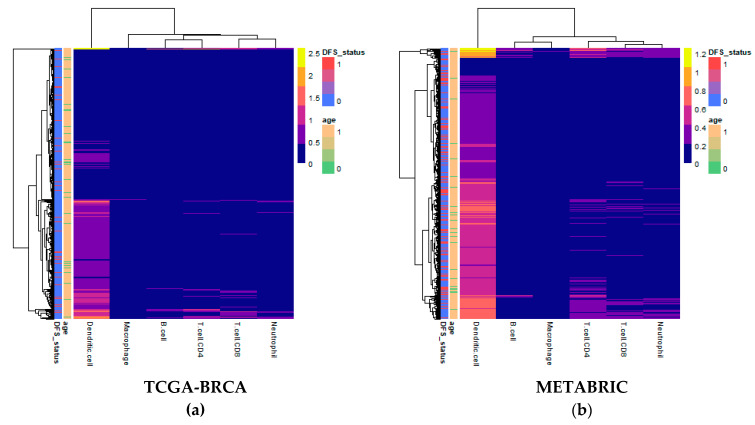
Heatmaps of abundance estimates for immune subsets predicted by computation deconvolution using the tumor immune estimation resource (TIMER) algorithm for the two cohorts: (**a**) The Cancer Genome Atlas (TCGA)-Breast Cancer (BRCA); and (**b**) Molecular Taxonomy of Breast Cancer International Consortium (METABRIC). Row and column dendrograms show clustering of cases and cell types, respectively, according to Euclidean distance.

Computational deconvolution of bulk gene expression data by the tumor immune estimation resource (TIMER) method, which used the constrained linear least-squares regression approach, allowed for abundance quantification of six immune cell types in each sample [19]. Differences in estimated immune cell abundance between age groups could not be distinguished by visual inspection of heatmaps for either cohort (Figure 1). The absolute differences in median between age groups in the Molecular Taxonomy of Breast Cancer International Consortium (METABRIC) cohort ranged only from 0.0065 for CD8+ T cells to 0.0715 for dendritic cells.

### 2.2. Immune Cell Type Abundance Significantly Associated with Disease-Free Survival

**Figure 2:**
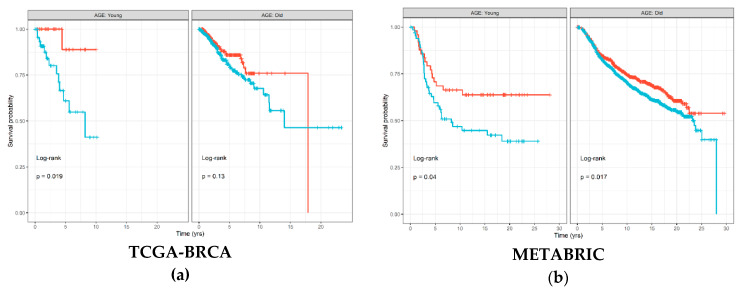
Disease-free survival Kaplan–Meier (KM) curve for: (**a**) TCGA-BRCA; and (**b**) METABRIC cohorts, grouped by age groups and stratified by high (red) and low (blue) CD8+ T cell levels estimated by TIMER and binarized by the maximally selected ranked statistics algorithm. Depicted *p*-values are from log-rank tests. p: *p-value*.

The Kaplan–Meier (KM) survival curve was used to associate abundance of each of the six immune cell types estimated by TIMER with disease-free survival (DFS) time and status separately for each age groups. Figure 2 shows that samples with high estimated CD8+ T cell abundance in The Cancer Genome Atlas (TCGA)-Breast Cancer (BRCA) cohort had significantly better prognosis (log-rank *p* = 0.019), which was also replicated in the METABRIC cohort (log-rank *p* = 0.04). Similar trends were also visible in the old age group; however, the differences in survival were less substantial between samples with high and low levels of CD8+ T cells. Figures S1 and S2 show results for all immune cell types from the TCGA-BRCA and METABRIC cohorts, respectively. For the other immune cell types, results were discordant between the two cohorts; however, macrophages seemed to consistently demonstrate little to no significant associations with DFS in the young age group in both the TCGA-BRCA (log-rank *p* = 0.94) and METABRIC (log-rank *p* = 0.28) cohorts.

**Figure 3:**
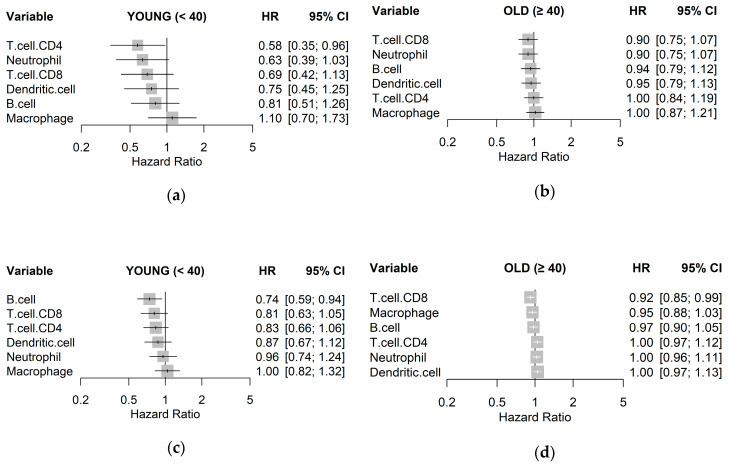
Unadjusted hazard ratios of each immune cell type quantified by TIMER, as individually estimated by univariable Cox regression models on disease-free survival for: (**a**) TCGA-BRCA young, (**b**) TCGA-BRCA old; (**c**) METABRIC young; and (**d**) METABRIC old cohorts with 95% confidence intervals (CIs).

To quantify associations with survival, a Cox proportional hazards regression model was fit for each immune cell type to estimate a hazard ratio (HR), visualized as a forest plot for each age group and cohort in Figure 3. The trend across both cohorts for the young age group was that higher estimates of immune cell types resulted in lower HR, albeit with little significance, with the exception of macrophages. This trend, however, was not observed in samples from the old age groups. In particular, CD8+ T cells were consistently associated with better clinical outcome in the young age group in both TCGA-BRCA (HR 0.69; *p* = 0.150) and METABRIC (HR 0.81; *p* = 0.110) cohorts, as compared to the old age group. High levels of CD4+ T cells were also associated with lower HR for the young age group in both TCGA-BRCA (HR 0.58; *p* = 0.150) and METABRIC (HR 0.83; *p* = 0.130) cohorts but not in the old age group. Abundance of macrophages was consistently shown as having little relationship with survival in all samples.

### 2.3. The Mutational Signature Characteristic of High TILs Differs across Age Groups

We investigated whether mutational burdens from particular mutational signatures were significantly different between the age groups in the TCGA-BRCA cohort by analyzing the associated whole-exome sequencing (WES) data. Table S1 shows that contributions from signature 12 were significantly higher in the young age group (Mann–Whitney U, MWU *p* = 0.00145), with a median fold change of 1.48, as well as signature 14 (MWU *p* = 0.0425), with a median fold change of 2.18. None of the signatures were found to be significantly higher in the old age group compared to the young. Mutation data associated with the METABRIC cohort from targeted sequencing of 172 genes were also analyzed; however, results were largely discordant with TCGA-BRCA data. Reconstruction accuracy between estimated mutational burden and actual represented by mean cosine similarity was lower in the METABRIC (0.538) compared to TCGA-BRCA (0.775) cohorts, likely due to differences in the total number of mutations captured from sequencing.

The presence and abundance of TILs, especially CD8+ T cells, have been frequently and consistently indicated as an important factor to consider for prognosis and treatment of breast cancer [8,11,18]. Therefore, we referred to the abundance of CD8+ T cells estimated by TIMER as a measure of TILs for subsequent analyses. For each age group independently, we investigated whether contributions from particular mutational signatures were significantly different between high and low CD8+ T cell groups as estimated by TIMER in the TCGA-BRCA cohort. None of the mutational signatures were associated with TIL levels in the young age group. However, in the old age group, mutational contributions from signature 1 (MWU *p* = 0.00504; fold change 1.23), signature 2 (MWU *p* = 0.0395; fold change 0.77), signature 17 (MWU *p* = 0.00319; fold change 0.99), signature 26 (MWU *p* = 0.0326; fold change 2.02), and signature 30 (MWU *p* = 0.0455; fold change 1.36) were significantly associated with TIL levels estimated by TIMER.

### 2.4. Gene Set Enrichment for High TILs

**Figure 4:**
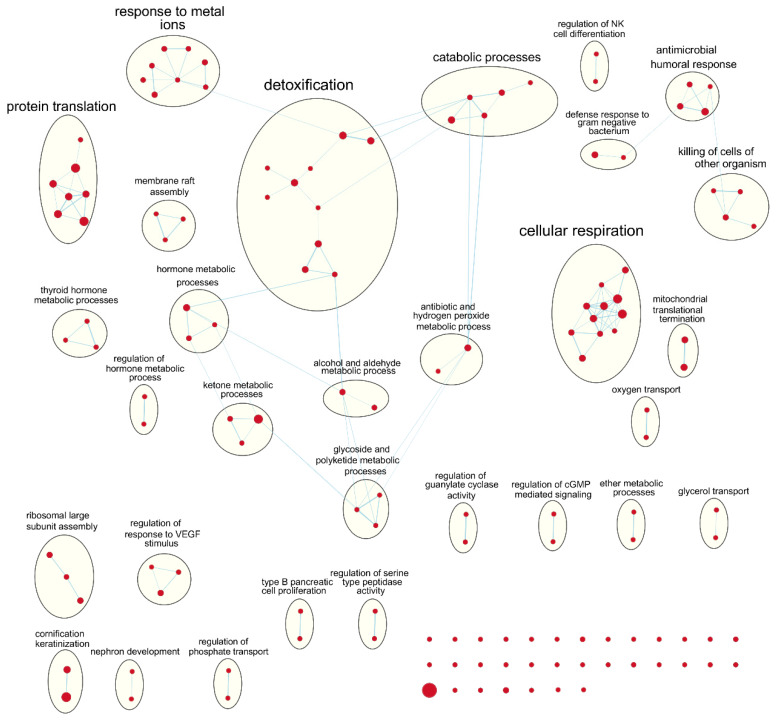
Enrichment map of results from preranked GSEA on the ranked gene list from the young TCGA-BRCA cohort. Nodes represent gene sets significant at FDR *q*-value < 0.25 and edges are drawn between nodes with similarity coefficient > 0.5. NK: natural killer; cGMP: cyclic guanosine monophosphate; VEGF: vascular endothelial growth factor.

**Figure 5:**
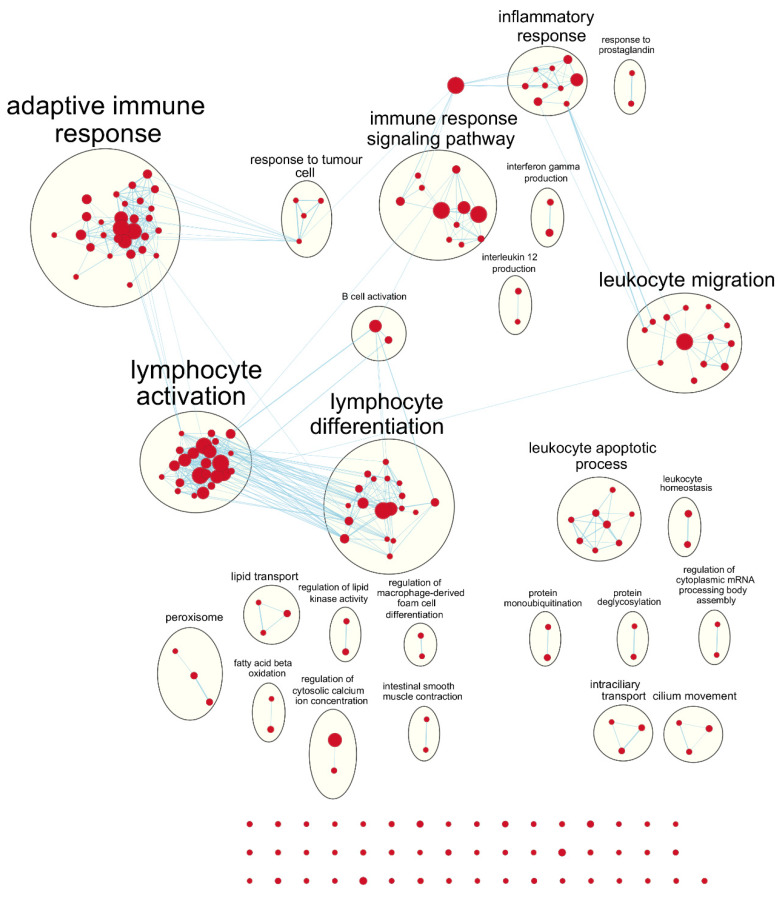
Enrichment map of results from preranked GSEA on the ranked gene list from the young METABRIC cohort. Nodes represent gene sets significant at FDR *q*-value < 0.25 and edges are drawn between nodes with similarity coefficient > 0.5. mRNA: messenger ribonucleic acid.

Following our results from survival analyses which demonstrated this pattern in the young age group but not in the old, we sought to validate TIMER estimates by examining which gene sets from the Gene Ontology project were enriched in samples with high TIL independently for each age group. Table 1 shows the number of gene sets enriched for genes positively associated with TIL at various significance levels. At false discovery rate (FDR) *q* < 0.05, there was one overlapping positively enriched gene set between TCGA-BRCA and METABRIC cohorts for each age group: “cotranslational protein targeting to membrane” in the young age group, and “cilium movement” in the old age group. Gene set enrichment analysis (GSEA) results from the young TCGA-BRCA cohort showed enrichment of gene sets related to the mitochondria and cellular respiration, among several others (Table S2). In contrast, in the young age group from the METABRIC cohort, many of the enriched gene sets were related to the adaptive immune response, most notably T cell proliferation, selection, and regulation of cytotoxicity (Table S3). In the old age group from both cohorts, many of the top positively enriched gene sets were related to cilium assembly, movement, and ciliary transport (Tables S4 and S5). For enrichment maps of enriched gene sets for the young patient group in each cohort, refer to Figure 4 and Figure 5.

## 3. Discussion

Despite several reports in various parts of the world echoing the conclusion that young age at diagnosis is an indication for poor prognosis of breast cancer [4,6,20,21], researchers have been unsuccessful in identifying significant biological differences between young and old breast cancer patients. For example, Anders et al. found a number of genes that were differentially expressed between younger and older breast cancer patients, but none remained significant after correcting for subtype and other clinicopathological features [3,5]. Our results are consistent with previous findings in that we were also unable to uncover significant differences in immune cell compositions estimated by gene expression deconvolution between young and old patients.

Various immune subsets should decline with age in a phenomenon known as immunosenescence [22]. The older patient population was also expected to have greater myeloid potential and lesser lymphocyte potential due to involution of the thymus [22]. These observations were not apparent in our analyses of differential immune profiles between age groups, which may be due to tumor-associated immune responses masking the global age-related changes in the immune system. Immunosenescence in women can also be attributed to deprivation of estrogen, which has immune-enhancing activities, during menopause [23]. In the datasets we used, 95.8% and 100% of the young patients (age at diagnosis <40) were pre-menopausal, whereas in the older patients (age at diagnosis ≥40) 20.3% and 16.5% were pre-menopausal in the TCGA-BRCA and METABRIC cohorts, respectively. Hence, menopausal state was a potential confounding variable in our study. Therefore, we fit a multiple linear regression model to further explore broader associations between estimated TIL, age at diagnosis (continuous variable), and menopausal state (categorical variable) (refer to Table S6). We found that TIL levels estimated by TIMER were significantly lower with older age at diagnosis (*p*-value: 0.0258 and 0.000282 for TCGA-BRCA and METABRIC cohorts, respectively). This finding is consistent with a recent meta-analysis exploring tumor-infiltrating lymphocytes and prognosis of early-stage triple-negative breast cancers [24]. However, menopausal state was not associated with TIL levels in both cohorts.

We found that specific immune subsets, in particular the CD8+ T cells, were significantly associated with disease-free survival in the young breast cancer patients but not in their older counterparts. This is in line with past studies that showed that higher infiltration of cytotoxic immune cells is indicative of better prognosis and greater odds of response to treatment [8,11]. Notably, Ali et al. showed that CD4+ and CD8+ T cells were more closely associated with favorable outcomes in ER-negative tumors than in ER-positive tumors [16]. Considering the fact that receptor status-negative tumors are much more common in the younger subset of breast cancer patients, our results are consistent with previous findings [4,7].

GSEA results showed that gene sets related to adaptive immune response and cytotoxic T cell processes were most obviously enriched in the young age group from the METABRIC cohort. On the other hand, gene sets related to the mitochondria and oxidative phosphorylation were positively enriched in the young age group from the TCGA-BRCA cohort. It may not be obvious, but mitochondria are important for T cell activation, proliferation, and differentiation because they are involved in processes such as immune synapse functions, production of reactive oxygen species, and various metabolic processes [25]. In the old age group from both cohorts, gene sets related to cilium assembly, movement, and ciliary transport were positively enriched for the high TIL phenotype, although with lower significance. Ciliary processes have recently been proposed to be involved in the formation of the immunological synapse between T cells and antigen-presenting cells, which is necessary for T cell activation and downstream functions [26,27]. This suggests that the TIMER algorithm may focus on different subset of genes for different datasets to provide estimates of immune cell composition.

Mutational signatures analysis showed that signatures 12 and 14 were significantly enriched in young compared to old breast cancer patients; however, the two signatures have not been previously associated with breast cancer [28,29]. Signature 12 was originally identified in liver cancer and is characterized by T > C substitutions with a transcriptional-strand-bias, which is indicative of being associated with transcription-coupled nucleotide excision repair [28]. Signature 14 was first identified in uterine cancer and is characterized by C > A and C > T substitutions, with a recent study suggesting that it is associated with microsatellite instability due to defects in mismatch repair [30]. However, our current knowledge on mutational signatures specific to breast cancer is likely reflective of the majority of breast cancer cases that are diagnosed in those over the age 40 (~93%). The landscape of mutational signatures specific to early onset breast cancer (~7%) has not been characterized previously. Further research is necessary to validate if the signatures previously not associated with breast cancer or novel signatures are responsible for mutations in the young subset of breast cancer patients. Within the young age group, none of the 30 single base substitution (SBS) signatures were significantly associated with TIL levels estimated by TIMER. However, within the old age group, mutational burdens from signatures 1, 26, and 30 were significantly higher in samples with high TIL levels, whereas signatures 2 and 17 were significantly associated with low TIL levels, although to varying degrees. Moreover, mutational signatures that were significantly associated with TIL levels in the old age group were all previously reported in breast cancer patients, which once again suggests that TIL levels estimated by TIMER may also be indicative of tumor purity within samples.

TIMER has been frequently used on RNA-seq data from the TCGA database, since it was originally developed on TCGA data to work with bulk RNA-seq data [19,31,32]. However, it must be noted that the reference gene sets used in TIMER were curated from microarray gene expression data. To our knowledge, we are the first to employ the method for analyzing microarray data from the METABRIC cohort. Microarray gene expression data suffers from artifacts, such as limited profiling of genes with very low expression levels or saturation at very high expression levels, which poses potential challenges for data analysis compared to bulk RNA-seq data [19,33]. However, authors of TIMER method have stated that both RNA-seq and microarray data may be used as input to estimate the abundance of immune cell types in the tumor microenvironment [19,33]. They showed that TIMER is capable of producing highly concordant estimates between RNA-seq and microarray gene expression data generated from the same tumor samples [19]. This demonstrated that the algorithm used for deconvolution, constrained least-square regression (Methods: Equation (1)), is robust regardless of input data type [19,33].

TIMER only provides estimates for six broad immune cell types: B cells, CD4+ T cells, CD8+ T cells, neutrophils, macrophages, and dendritic cells [19]. This can be advantageous because limiting number of cell types interrogated to those that are linearly separable prevents instability of estimates due to statistical co-linearity between cell types with very similar gene expression [19,32]. However, this is at the expense of resolution of the output. Even though they may originate from the same progenitor cell, different immune cell types and states can play vastly different roles within the tumor tissue; for example, M0 and M2 macrophages have traditionally been associated with pro-tumor responses whereas M1 macrophages have been associated with anti-tumor responses [7,18]. Our results from TIMER that found macrophages consistently insignificant to clinical outcomes in both cohorts may stem from the fact that TIMER cannot distinguish between the different activation states of macrophages. Despite its limitations, we were able to use TIMER as a tool to estimate the composition of immune cells within breast tumor samples and find specific immune subsets that showed significant trends with regards to disease-free survival in the young age group of breast cancer patients but not in the old.

## 4. Materials and Methods

### 4.1. Data

From The Cancer Genome Atlas (TCGA) database, gene-level RNA-seq expression data of patients with primary breast cancer (BRCA) were downloaded from Genomic Data Commons (GDC) portal (https://portal.gdc.cancer.gov/) through TCGA-Assembler 2 [12,34]. The gene expression data had previously been processed by the RNAseqV2 pipeline, providing estimated counts and scaled estimate values from RNA-seq by Expectation Maximization (RSEM) [35]. Scaled estimates were converted to transcripts per million (TPM) values by multiplying by one million, resulting in a bulk RNA-seq dataset of 20,501 genes from 1095 breast tumor samples. We used RSEM-processed TPM measure because it was used previously to develop the TIMER method [19]. Mutations data from whole-exome sequencing (WES) and clinical data associated with the TCGA-BRCA cohort were downloaded from the cBioPortal for Cancer Genomics (https://www.cbioportal.org/) [36]. Samples from male patients (*n* = 12) were excluded from analyses, as well as any samples without associated age or survival data, resulting in final sample size of 989. There are inconsistencies in age thresholds used to define early onset breast cancer [1,4,5,6]. Here, we used a relatively conservative, but commonly used threshold (age at diagnosis = 40) to define it. Hence, of the 989 samples, 70 were identified as young/early-onset (age <40), and 919 were identified as old (age ≥40).

From the Molecular Taxonomy of Breast Cancer International Consortium (METABRIC) cohort, gene-level microarray expression data and associated clinical data were downloaded from cBioPortal [13,36]. The gene expression data had previously been processed to log2 intensity values as described in the original publication [13]. Samples without associated age or survival data were removed, resulting in bulk microarray dataset of 24,360 genes from 1903 breast tumor samples, of which 116 were young and 1787 were old.

### 4.2. Immune Cell Type Deconvolution

Tumor immune estimation resource (TIMER) was used to deconvolute bulk gene expression data to estimate immune cell abundance [19]. In brief, most deconvolution algorithms see bulk transcriptome as a mixture where one gene of the mixture is a linear combination of that gene expressed across different cell types, weighted by the proportions of those cell types [15]. This can be represented by a simple Equation of a linear model:

(1) $$y_{i}={\hat{\beta }}_{i1}x_{i1}+{\hat{\beta }}_{i2}x_{i2}+{\hat{\beta }}_{i3}x_{i3}+\cdots +{\hat{\beta }}_{ik}x_{ik}\;,$$

where $y_{i}$ is the gene expression level of a single gene i in the mixture data of n number of genes, $x_{1}$, $x_{2}$, …, $x_{k}$ are gene expressions of the one gene across k cell types, and ${\hat{\beta }}_{1}$, ${\hat{\beta }}_{2}$, …, ${\hat{\beta }}_{k}$ are relative fractions of those cell types [15,19]. Given a signature expression matrix X, consisting of n rows for number of genes and k columns for number of cell types, the deconvolution algorithm produces estimates of cell fractions ($\hat{\beta }$). Specifically, TIMER utilizes constrained linear regression with non-negativity constraint on cell fractions to estimate the abundance of six immune cell types: B cells, CD4+ T cells, CD8+ T cells, neutrophils, macrophages, and dendritic cells [19]. For settings, the sample tumor type was set to “BRCA” for breast cancer, and the default reference data and gene set were used for both cohorts.

The TIMER method was available for quick implementation through the immunedeconv package [15,19]. Expression data with rows as genes and columns as samples was provided to the algorithm as input, with which the algorithm estimated abundance of six immune cell types. Results were visualized as heatmaps and violin plots using the pheatmap [37] and ggpubr [38] packages, respectively.

### 4.3. Survival Analyses

Kaplan–Meier (KM) survival curves were visualized using survival [39] and survminer packages [40]. Maximally selected rank statistics (maxstat) as implemented in the survminer package was used to determine the optimal cut-off to binarize the immune cell abundance estimates from TIMER into “high” and “low” groups which produces the maximum log-rank statistic on disease-free survival (DFS) [41].

Cox regression was used to compute hazard ratios for each of the six immune cell types for which abundance was estimated by TIMER. Analyses were conducted separately for each age group to consider the potential violation of the Cox proportional-hazards assumption [16]. Similar to previous approach by Ali et al., estimates of immune cell abundance were converted to quartiles from 1–4 [16]. Results were visualized using the meta package [42].

### 4.4. Single Base Substitution Mutational Signatures

From the TCGA-BRCA cohort, 981 samples had mutation data available. Number of mutations for each of 96 possible single base substitution (SBS) types were summed using deconstructSigs package [43]. With the resulting matrix of SBS counts by samples, contributions of each of the 30 SBS signatures from the Catalogue of Somatic Mutations in Cancer (COSMIC) database [44] were estimated for each sample using the R package MutationalPatterns [45]. Instead of excluding SBS signatures previously not extracted from breast cancer cohorts, all 30 signatures were incorporated in our analysis because the young subset of breast cancer patients is largely underrepresented in many cohorts that have been used to extract those signatures [27]. Samples with pairwise cosine similarity between the reconstructed mutation counts matrix and the original input less than 0.5 were excluded (*n* = 23). Samples without gene expression or relevant clinical data were also excluded, resulting in 858 samples, of which 57 were young and 801 were old. Estimated contribution from each signature was compared by Mann–Whitney U (MWU) test between young and old age groups [27].

### 4.5. Gene Set Enrichment Analysis

To find genes positively associated with CD8+ T cell abundance estimated by TIMER in each cohort, simple linear regression models were fit for expression of each gene separately, with expression values treated as the continuous predictor variable and TIMER estimates as the continuous outcome variable. Models were fit separately for each age group, and the t-statistic value for each of the resulting regression coefficients were calculated as a measure of association to the CD8+ T cell abundance estimates. The list of genes ordered by decreasing t-statistic values for each age group was used as input for pre-ranked gene set enrichment analysis (GSEA) on the C5:BP (Gene Ontology biological processes) gene sets downloaded from the Molecular Signatures Database (MSigDB; http://software.broadinstitute.org/gsea/msigdb/index.jsp) [46,47]. Gene sets of size < 2 and > 500 were excluded, resulting in total of 7094 gene sets evaluated in the analysis. Results from GSEA were visualized by Cytoscape version 3.7.2 [48] using the Enrichment Map plugin [49]. Enriched gene sets with FDR q value < 0.25 were represented as nodes and any overlaps > 0.3 between nodes were represented as edges in the resulting network diagram. Nodes were grouped together and labelled by the clusterMaker2 [50], AutoAnnotate [51], and WordCloud [52] plugins, and resulting annotations were manually corrected.

### 4.6. Data Analysis Software

All analyses and visualizations were performed in R Project for Statistical Computing version 3.6.1 [53] and RStudio version 1.2.1335(Boston, MA, United States) [54]. Unless otherwise noted, all statistical analyses were conducted using the stats package [53], and two-sided *p* < 0.05 was considered significant.

## 5. Conclusions

In summary, we determined that a particular immune cell type, the cytotoxic CD8+ T cell, was significantly associated with disease-free survival in young breast cancer patients under the age 40, but not in their older counterparts. Furthermore, our analyses showed that single base substitution mutational signatures 12 and 14 were significantly enriched in the young patient group compared to the old, that were not previously associated with breast cancer. We also highlighted some potential limitations of our data and methods, especially with TIMER and its lack of resolution. Nonetheless, our work suggests that the underlying biological differences may stem from more abstract relationships involving multiple levels of tumor physiology and not age alone.

## Figures and Tables

**Table 1:** Number of positively enriched gene sets at different significance levels for each age group in each cohort from preranked gene set enrichment analysis (GSEA).

Age Group	Dataset	Number of Features	Number of Positive Gene Sets	Nominal *p* < 0.01	FDR *q* < 0.25	FDR *q* < 0.05	Number of Overlaps at FDR *q* < 0.05
Young	TCGA-BRCA	19,879	1370	126	135	33	1
Young	METABRIC	24,360	3765	246	204	30	
Old	TCGA-BRCA	20,201	2801	42	3	0	1
Old	METABRIC	24,360	3793	95	8	1	

^1^ “Number of features” denotes the number of genes in the ranked gene list used as input for the analyses.

## Supplementary Materials

The following are available online at https://www.mdpi.com/2072-6694/12/5/1076/s1. Figure S1: 10-year disease-free survival Kaplan–Meier curves for TCGA-BRCA cohort; Figure S2: 10-year disease-free survival Kaplan–Meier curves for the METABRIC cohort; Table S1: Differences in mutational burden from 30 SBS signatures from COSMIC between young and old age groups in TCGA-BRCA cohort; Table S2: Preranked GSEA results for the gene list ranked by correlation with TIL levels in the young age group from TCGA-BRCA cohort; Table S3: Preranked GSEA results for the gene list ranked by correlation with TIL levels in the young age group from the METABRIC cohort; Table S4: Preranked GSEA results for the gene list ranked by correlation with TIL levels in the old age group from the TCGA-BRCA cohort; Table S5: Preranked GSEA results for the gene list ranked by correlation with TIL levels in the old age group from the METABRIC cohort; Table S6: Coefficients and statistical results of multiple linear regression between the response variable: TIL level estimated by TIMER, and predictor variables: age at diagnosis, and menopausal state.
