# Supplementary material for: Tumor-Infiltrating CD8 T Cells Predict Clinical Breast Cancer Outcomes in Young Women
Source: Cancers (Basel). 2020 Apr 26;12(5):1076. doi: 10.3390/cancers12051076 (PMC7281139; doi:10.3390/cancers12051076)
Supplement: Supplementary file 1 [file cancers-12-01076-s001.pdf]

cancers

MDPI

Article

Tumor-Infiltrating CD8 T Cells Predict Clinical Breast Cancer Outcomes in Young Women

Yong Won Jin and Pingzhao Hu

Supplemental Material

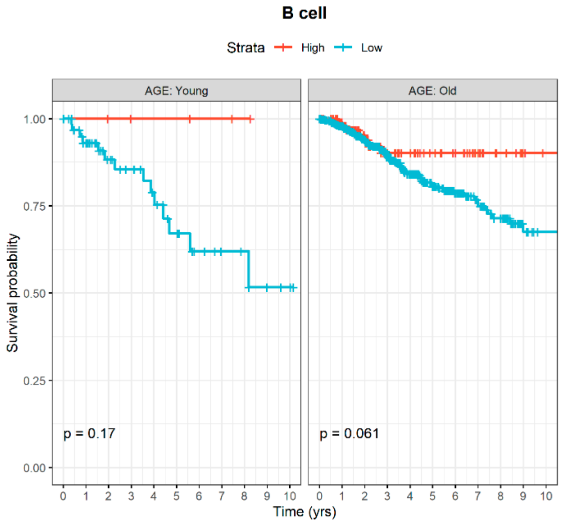

(**a**)

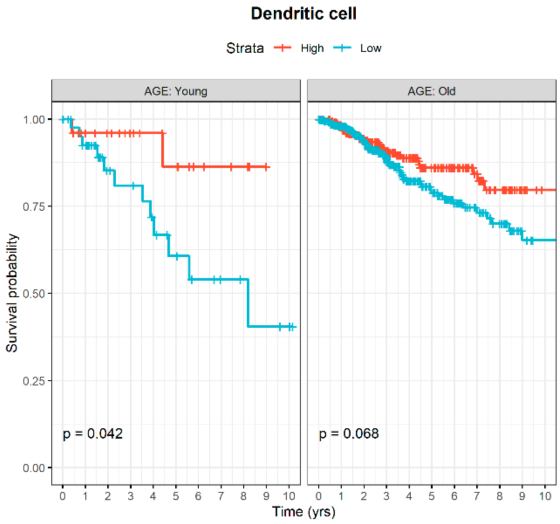

(**b**)

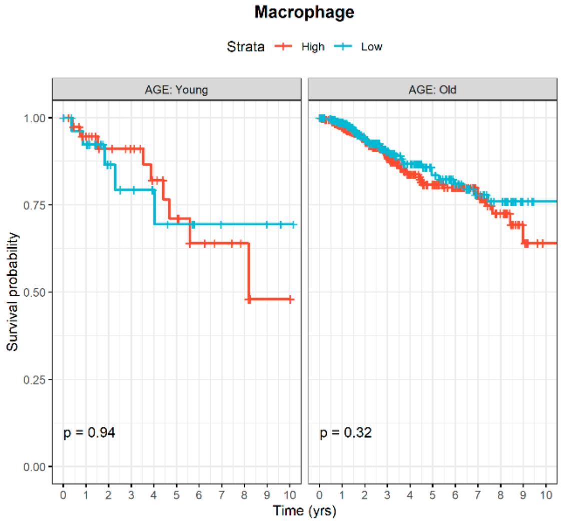

(**c**)

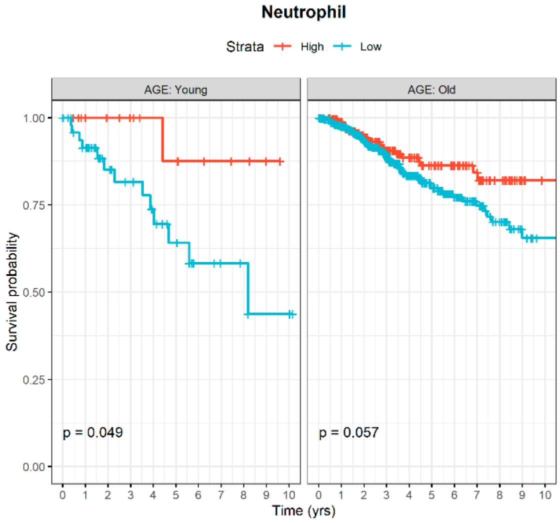

(**d**)

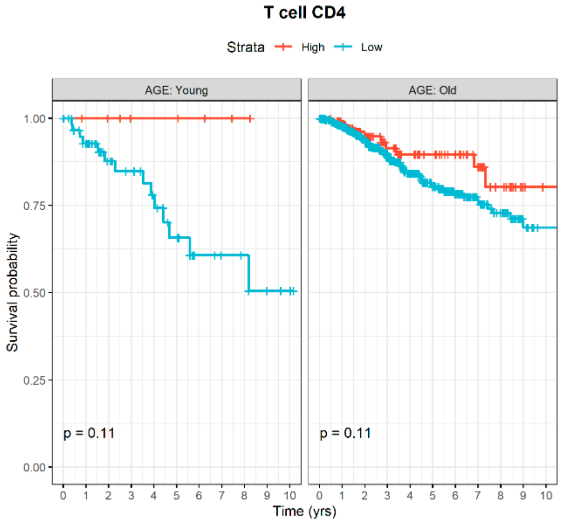

(**e**)

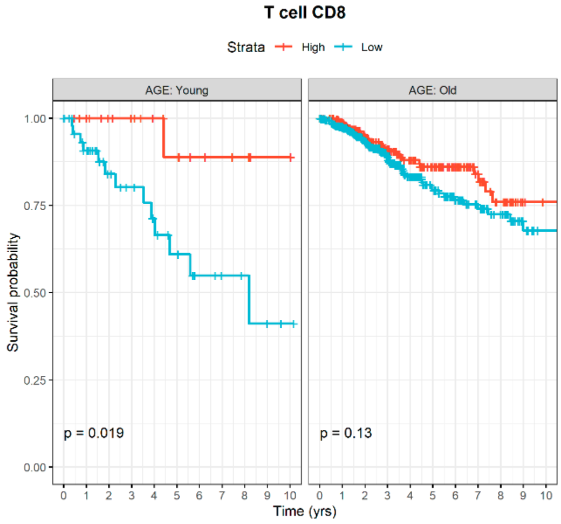

(**f**)

Figure S1. 10- year disease-free survival KM curve for TCGA-BRCA cohort, grouped by age groups and stratified by high (red) and low (blue) levels of six immune cell types estimated by TIMER: (**a**) B cell; (**b**) dendritic cell; (**c**) macrophage; (**d**) neutrophil; (**e**) CD4+ T cell; and (**f**) CD8+ T cell. Immune cell levels were binarized by maxstat algorithm. Depicted *p*-values are from log-rank tests.

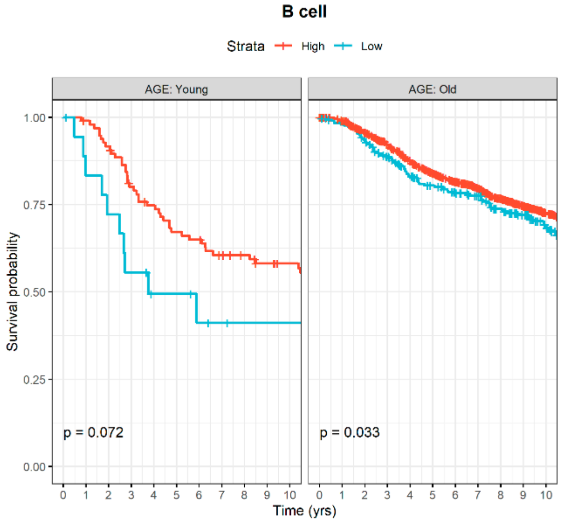

(**a**)

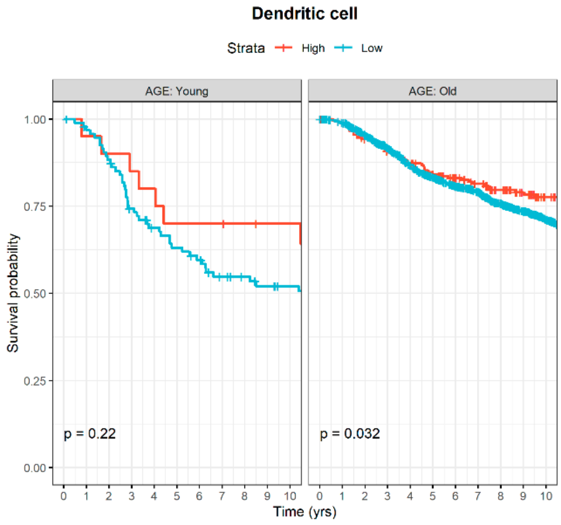

(**b**)

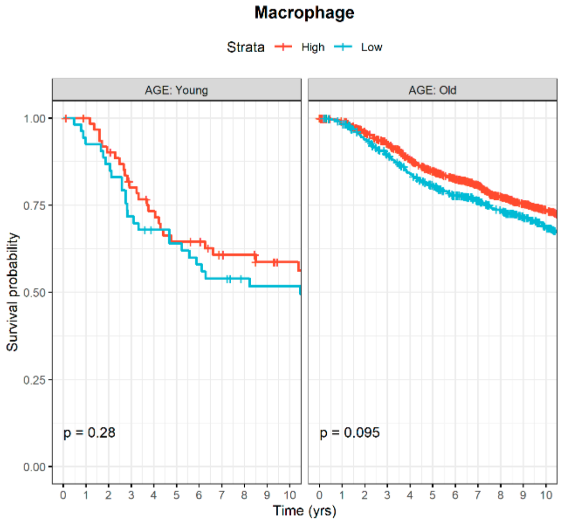

(c)

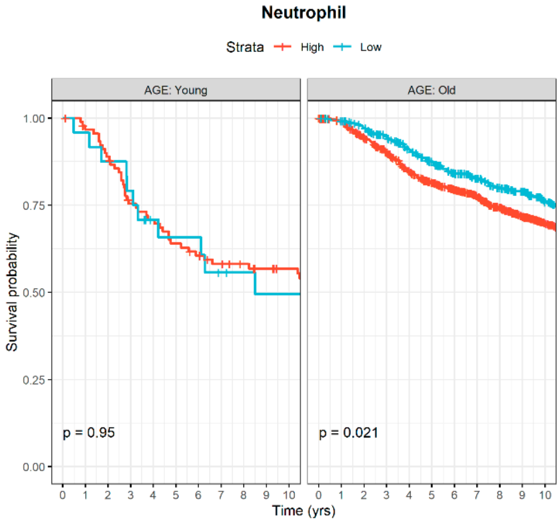

(d)

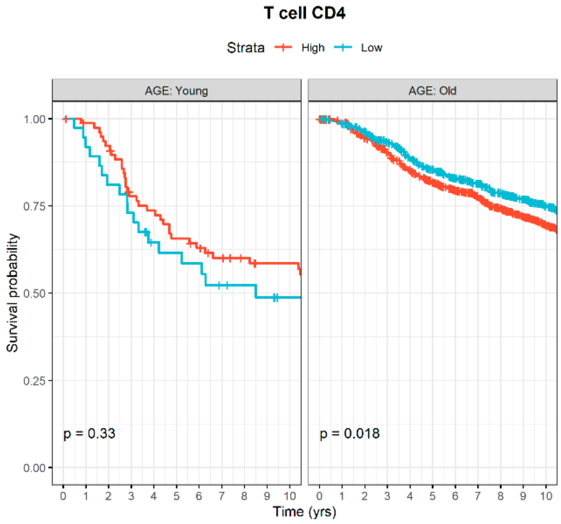

(e)

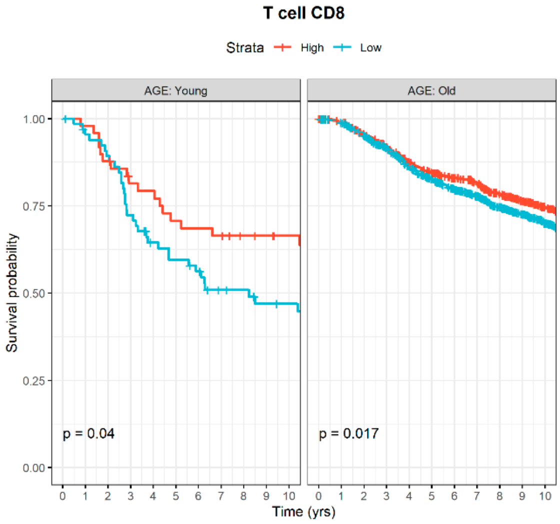

(f)

Figure S2. 10- year disease-free survival KM curve for METABRIC cohort, grouped by age groups and stratified by high (red) and low (blue) levels of six immune cell types estimated by TIMER: (**a**) B cell; (**b**) dendritic cell; (**c**) macrophage; (**d**) neutrophil; (**e**) CD4+ T cell; and (**f**) CD8+ T cell. Immune cell levels were binarized by maxstat algorithm. Depicted *p*-values are from log-rank tests.

Table S1. Differences in mutational burden from 30 SBS signatures from COSMIC between young and old age groups in the TCGA-BRCA cohort.

Signature	*n*	*p*	YOUNG mutational burden	OLD mutational burden	Fold change
1	767	0.217108	10.34976	10.59553	0.976804901
2	549	0.325358	3.888289	3.996138	0.973011829
3	343	0.690164	17.75103	8.407852	2.111244358
4	211	0.901561	3.976115	4.530104	0.87770942
5	24	0.185634	NA	2.53213	NA
6	374	0.335386	4.918544	5.84488	0.841513243
7	441	0.367437	3.826739	3.4218	1.118341061
8	120	0.128638	5.379963	3.999362	1.345205173
9	42	0.417925	1.868344	0.93043	2.00804291
10	426	0.068364	1.563409	2.486163	0.628843997

11	174	0.379684	2.325416	2.157148	1.078005214
12	149	0.001447	3.469254	2.341764	1.481470608
13	567	0.515672	3.193183	3.748332	0.851894393
14	88	0.042543	3.95495	1.810553	2.184388059
15	283	0.256524	2.316104	4.389657	0.527627423
16	164	0.832406	2.515793	3.27996	0.767019314
17	276	0.944968	3.159945	1.774596	1.780655868
18	214	0.192837	3.529686	2.599131	1.358025173
19	130	0.479199	2.194205	3.119958	0.703280388
20	229	0.964294	2.374497	2.731509	0.86929863
21	236	0.646418	1.912902	1.848762	1.034693208
22	304	0.76474	1.815304	1.421824	1.276743206
23	151	0.84864	0.940823	1.727247	0.544694984
24	306	0.285911	4.629516	3.332312	1.389280494
25	25	0.767103	8.925052	2.703811	3.300916033
26	144	0.757082	1.104366	2.569928	0.429726538
27	126	0.705574	0.16551	0.80792	0.204859272
28	157	0.283617	0.443062	1.248088	0.354992647
29	235	0.676288	2.515517	3.203889	0.785144874
30	139	0.102988	1.305804	2.927002	0.446123188

[1] n shows number of samples with mutational burden greater than zero from that particular signature.

[2] p denotes the p value from MWU test between young and old groups.

[3] Subsequent columns show the median mutational burden for each signature in each age group.

Table S2. GSEA preranked results for gene list ranked by correlation with TIL levels in the young age group from TCGA-BRCA cohort.

NAME	SIZE[1]	ES[2]	NES[3]	NOM p[4]	FDR q[5]	FWER p[6]
GO_COTRANSLATIONAL_PROTEIN_TARGETING_TO_MEMBRANE	97	0.544279	2.847659	0	0	0
GO_ESTABLISHMENT_OF_PROTEIN_LOCALIZATION_TO_ER	110	0.492764	2.655034	0	4.77E-04	0.001
GO_STRESS_RESPONSE_TO_METAL_ION	15	0.780708	2.597907	0	6.39E-04	0.002
GO_ATP_SYNTHESIS_COUPLED_ELECTRON_TRANSPORT	79	0.518184	2.561809	0	9.47E-04	0.004
GO_COFACTOR_CATABOLIC_PROCESS	64	0.522318	2.551812	0	7.57E-04	0.004
GO_RESPIRATORY_ELECTRON_TRANSPORT_CHAIN	97	0.47257	2.499912	0	0.001425	0.009
GO_DETOXIFICATION	112	0.454115	2.460509	0	0.001641	0.012
GO_KILLING_OF_CELLS_OF_OTHER_ORGANISM	54	0.506229	2.428837	0	0.00192	0.016
GO_HYDROGEN_PEROXIDE_CATABOLIC_PROCESS	32	0.60728	2.420802	0	0.001814	0.017
GO_CORNIFICATION	111	0.445246	2.417347	0	0.001829	0.019
GO_PROTEIN_LOCALIZATION_TO_ER	134	0.425255	2.323629	0	0.005919	0.065
GO_OXIDATIVE_PHOSPHORYLATION	101	0.43471	2.318714	0	0.005665	0.068

GO_ANTIBIOTIC_CATABOLIC_PROCESS	50	0.484244	2.300764	0	0.006411	0.08
GO_NUCLEAR_TRANSCRIBED_MRNA_CATABOLIC_PROCESS_NONSENSE_MEDIATED_DECAY	114	0.420275	2.282805	0	0.007047	0.095
GO_GAS_TRANSPORT	19	0.628352	2.273192	0	0.00773	0.112
GO_ELECTRON_TRANSPORT_CHAIN	163	0.405188	2.223335	0	0.013208	0.197
GO_REGULATION_OF_SERINE_TYPE_PEPTIDASE_ACTIVITY	9	0.771998	2.216882	0	0.01328	0.21
GO_CELLULAR_RESPONSE_TO_ZINC_ION	21	0.606071	2.19978	0	0.016612	0.269
GO_KERATINIZATION	178	0.377058	2.185627	0	0.018429	0.303
GO_ACYLGLYCEROL_ACYL_CHAIN_REMODELING	7	0.83337	2.18484	0	0.017607	0.305
GO_MITOCHONDRIAL_ELECTRON_TRANSPORT_UBIQUINOL_TO_CYTOCHROME_C	12	0.660834	2.147358	0	0.024977	0.419
GO_C21_STEROID_HORMONE_METABOLIC_PROCESS	38	0.499533	2.134394	0	0.027856	0.466
GO_AEROBIC_ELECTRON_TRANSPORT_CHAIN	16	0.623273	2.120486	0	0.031017	0.517
GO_PEPTIDE_CROSS_LINKING	53	0.45384	2.110602	0	0.033145	0.563
GO_NEGATIVE_REGULATION_OF_SERINE_TYPE_PEPTIDASE_ACTIVITY	7	0.814052	2.101609	0	0.034936	0.601
GO_MITOCHONDRIAL_ELECTRON_TRANSPORT_NADH_TO_UBIQUINONE	45	0.480546	2.094323	0	0.03611	0.631
GO_RIBOSOMAL_LARGE_SUBUNIT_ASSEMBLY	28	0.537186	2.089743	0	0.036627	0.645
GO_PROGESTERONE_METABOLIC_PROCESS	10	0.710203	2.081615	0	0.038603	0.676
GO_OXYGEN_TRANSPORT	15	0.632096	2.078758	0	0.038627	0.691
GO_XENOBIOTIC_METABOLIC_PROCESS	107	0.380285	2.060239	0	0.045989	0.756
GO_BENZENE_CONTAINING_COMPOUND_METABOLIC_PROCESS	11	0.673088	2.057808	0	0.045593	0.765
GO_CELLULAR_DETOXIFICATION	100	0.384201	2.056978	0	0.0445	0.769
GO_FORMATION_OF_CYTOPLASMIC_TRANSLATION_INITIATION_COMPLEX	14	0.628908	2.050842	0.004255	0.04569	0.792

[1] Size indicates number of genes in the gene set.

[2] ES is the enrichment score.

[3] NES is the ES value normalized by size.

[4] NOM p denotes nominal p value

[5] FDR q denotes false discovery rate q-value. Only gene sets with FDR q-value < 0.05 are shown.

[6] FWER p denotes family-wise error rate p-value.

Table S3. GSEA preranked results for gene list ranked by correlation with TIL levels in the young age group from METABRIC cohort.

NAME	SIZE[1]	ES[2]	NES[3]	NOM *p*[4]	FDR *q*[5]	FWER *p*[6]
GO_ALPHA_BETA_T_CELL_PROLIFERATION	28	0.66127	2.348763	0	0.001097	0.001
GO_B_CELL_RECEPTOR_SIGNALING_PATHWAY	53	0.57866	2.300078	0	0.002778	0.005
GO_NEGATIVE_T_CELL_SELECTION	12	0.838532	2.254999	0	0.005192	0.014
GO_DENDRITIC_CELL_MIGRATION	24	0.662275	2.224096	0	0.007778	0.028
GO_INACTIVATION_OF_MAPK_ACTIVITY	24	0.659955	2.221114	0	0.006223	0.028
GO_ADAPTIVE_IMMUNE_RESPONSE	350	0.396757	2.159699	0	0.01592	0.084
GO_POSITIVE_T_CELL_SELECTION	32	0.588408	2.137951	0	0.020617	0.122
GO_PROTEIN_ACTIVATION_CASCADE	25	0.618532	2.136991	0	0.018177	0.123
GO_REGULATION_OF_B_CELL_RECEPTOR_SIGNALING_PATHWAY	24	0.640197	2.130815	0	0.017994	0.137
GO_REGULATION_OF_ANTIGEN_RECEPTOR_MEDIATED_SIGNALING_PATHWAY	53	0.522912	2.129687	0	0.01653	0.14
GO_POSITIVE_REGULATION_OF_ALPHA_BETA_T_CELL_PROLIFERATION	16	0.711918	2.125998	0	0.01563	0.145
GO_LYMPHOCYTE_COSTIMULATION	54	0.521161	2.089008	0	0.025422	0.24
GO_REGULATION_OF_CYTOPLASMIC_MRNA_PROCESSING_BODY_ASSEMBLY	8	0.870165	2.087923	0	0.024146	0.246
GO_T_CELL_SELECTION	44	0.542041	2.079368	0	0.024476	0.263
GO_THYMIC_T_CELL_SELECTION	20	0.635614	2.070601	0	0.025954	0.293
GO_LYMPHOCYTE_DIFFERENTIATION	312	0.386417	2.06839	0	0.024889	0.299
GO_POSITIVE_THYMIC_T_CELL_SELECTION	12	0.743443	2.056719	0.002024	0.027521	0.339
GO_REGULATION_OF_T_CELL_MEDIATED_CYTOTOXICITY	28	0.585715	2.043598	0	0.032096	0.406
GO_REGULATION_OF_LEUKOCYTE_APOPTOTIC_PROCESS	76	0.465984	2.031467	0	0.035965	0.463
GO_ANTIGEN_RECEPTOR_MEDIATED_SIGNALING_PATHWAY	216	0.388313	2.01726	0	0.041657	0.53
GO_PROTEIN_DEGLYCOSYLATION	25	0.592343	2.01484	0	0.041258	0.543
GO_POSITIVE_REGULATION_OF_LEUKOCYTE_CELL_CELL_ADHESION	197	0.398304	2.011936	0	0.040939	0.555
GO_POSITIVE_REGULATION_OF_T_CELL_MEDIATED_CYTOTOXICITY	21	0.616534	2.011214	0	0.039835	0.558
GO_IMMUNE_RESPONSE_REGULATING_CELL_SURFACE_RECEPTOR_SIGNALING_PATHWAY	355	0.364463	2.008047	0	0.039702	0.576
GO_LEUKOCYTE_PROLIFERATION	262	0.381872	2.003348	0	0.040511	0.599
GO_POSITIVE_REGULATION_OF_ADAPTIVE_IMMUNE_RESPONSE	88	0.447851	1.994216	0	0.044336	0.644

GO_HEMOGLOBIN_METABOLIC_PROCESS	5	0.939277	1.99229	0	0.043678	0.651
GO_POSITIVE_REGULATION_OF_CELL_ACTIVATION	289	0.374459	1.990124	0	0.04311	0.658
GO_URONIC_ACID_METABOLIC_PROCESS	9	0.788019	1.987935	0	0.042694	0.665
GO_ESTABLISHMENT_OF_LYMPHOCYTE_POLARITY	10	0.747246	1.974582	0	0.048517	0.725

[1] Size indicates number of genes in the gene set.

[2] ES is the enrichment score.

[3] NES is the ES value normalized by size.

[4] NOM *p* denotes nominal *p* value

[5] FDR *q* denotes false discovery rate *q*-value. Only gene sets with FDR *q*-value < 0.05 are shown.

[6] FWER *p* denotes family-wise error rate *p*-value.

Table S4. GSEA preranked results for gene list ranked by correlation with TIL levels in the old age group from TCGA-BRCA cohort.

NAME	SIZE[1]	ES[2]	NES[3]	NOM p[4]	FDR q[5]	FWER p[6]
GO_INTRACILIARY_TRANSPORT	49	0.531478	2.095876	0	0.198538	0.211
GO_CILIUM_MOVEMENT	45	0.549711	2.084047	0	0.118814	0.247
GO_OUTFLOW_TRACT_SEPTUM_MORPHOGENESIS	27	0.599063	2.020211	0	0.21298	0.538

[1] Size indicates number of genes in the gene set.

[2] ES is the enrichment score.

[3] NES is the ES value normalized by size.

[4] NOM p denotes nominal p value

[5] FDR q denotes false discovery rate q-value. Only gene sets with FDR q-value < 0.05 are shown.

[6] FWER p denotes family-wise error rate p-value.

Table S5. GSEA preranked results for gene list ranked by correlation with TIL levels in the old age group from METABRIC cohort.

NAME	SIZE[1]	ES[2]	NES[3]	NOM p[4]	FDR q[5]	FWER p[6]
GO_REGULATION_OF_CILIUM_ASSEMBLY	44	0.605123	2.31246	0	0.006294	0.006
GO_HEPATOCYTE_GROWTH_FACTOR_RECEPTOR_SIGNALING_PATHWAY	16	0.70647	2.066047	0	0.135248	0.228
GO_EPITHELIAL_CILIUM_MOVEMENT	16	0.675739	2.020608	0.001938	0.179515	0.399
GO_CILIARY_BASAL_BODY_PLASMA_MEMBRANE_DOCKING	80	0.465679	2.0138	0	0.151112	0.436
GO_HYALURONAN_BIOSYNTHETIC_PROCESS	12	0.743555	1.994188	0.001862	0.16514	0.549
GO_NEGATIVE_REGULATION_OF_MRNA_PROCESSING	28	0.57346	1.963609	0	0.22187	0.717
GO_POSITIVE_REGULATION_OF_CILIUM_ASSEMBLY	20	0.63506	1.96157	0.001855	0.195556	0.724
GO_CILIUM_MOVEMENT	40	0.520157	1.944506	0	0.207414	0.791

[1] Size indicates number of genes in the gene set.

[2] ES is the enrichment score.

[3] NES is the ES value normalized by size.

[4] NOM p denotes nominal p value

[5] FDR q denotes false discovery rate q-value. Only gene sets with FDR q-value < 0.05 are shown.

[6] FWER p denotes family-wise error rate p-value.

Table S6. Coefficients and statistical results of multiple linear regression between the response variable: TIL level estimated by TIMER; and predictor variables: age at diagnosis, and menopausal state.

<table>
<tr><th colspan="6">TIL ~ age_at_diagnosis + menopausal_state[1]</th></tr>
<tr><td rowspan="7">TCGA-BRCA</td><td colspan="5">Coefficients:</td></tr>
<tr><td></td><td>Estimate</td><td>Std. Error</td><td>t value</td><td>Pr(>|t|)</td></tr>
<tr><td>(Intercept)</td><td>0.2579713</td><td>0.0354456</td><td>7.278</td><td>1.09e-12</td></tr>
<tr><td>age_at_diagnosis</td><td>-0.0016823</td><td>0.0007642</td><td>-2.201</td><td>0.0281</td></tr>
<tr><td>menopausal_state = “Peri”</td><td>-0.0599772</td><td>0.0364901</td><td>-1.644</td><td>0.1008</td></tr>
<tr><td>menopausal_state = “Post”</td><td>0.0210642</td><td>0.0224287</td><td>0.939</td><td>0.3480</td></tr>
<tr><td colspan="5">F-statistic: 2.891 on 2 and 590 DF, p-value: 0.03151</td></tr>
<tr><td rowspan="6">METABRIC</td><td colspan="5">Coefficients:</td></tr>
<tr><td></td><td>Estimate</td><td>Std. Error</td><td>t value</td><td>Pr(>|t|)</td></tr>
<tr><td>(Intercept)</td><td>0.1474797</td><td>0.0094865</td><td>15.546</td><td>< 2e-16</td></tr>
<tr><td>age_at_diagnosis</td><td>-0.0007406</td><td>0.0002036</td><td>-3.638</td><td>0.000282</td></tr>
<tr><td>menopausal_state = “Post”</td><td>0.0064385</td><td>0.0064213</td><td>1.003</td><td>0.316143</td></tr>
<tr><td colspan="5">F-statistic: 9.864 on 2 and 1900 DF, p-value: 5.474e-05</td></tr>
</table>

[1] Menopausal state is a categorical variable with three levels (“Pre”, “Peri”, and “Post”) provided for TCGA-BRCA and two levels (“Pre”, and “Post”) provided for METABRIC data. For both cohorts, the first level (“Pre”) was used as reference.

© 2020 by the authors. Submitted for possible open access publication under the terms and conditions of the Creative Commons Attribution (CC BY) license (http://creativecommons.org/licenses/by/4.0/).
